# Bulbocavernosus Reflex Monitoring During Intramedullary Conus Tumor Surgery

**DOI:** 10.7759/cureus.7233

**Published:** 2020-03-10

**Authors:** Kathryn Overzet, Faisal R Jahangiri, Robert Funk

**Affiliations:** 1 Neurophysiology, Axis Neuromonitoring, Richardson, USA; 2 Neurophysiology, Global Innervation, Dallas, USA; 3 Neurosurgery, United Regional Health Care System, Wichita Falls, USA

**Keywords:** conus medullaris, bulbocavernosus reflex, transcranial electrical motor evoked potentials, electromyography, neurophysiology, spine tumor, ionm, urethral sphincter monitoring, bcr, intraoperative neurophysiological monitoring

## Abstract

A T10 to L2 spinal cord tumor exploration and biopsy was performed with intraoperative neurophysiological monitoring (IONM) on a 75-year-old male diagnosed with an intradural intramedullary appearing spinal cord lesion with no other lesions in the central nervous system, chest, abdomen or pelvis.

Intraoperative neurophysiology consisted of transcranial electrical motor evoked potentials (TCeMEPs), somatosensory evoked potentials (SSEPs), triggered and spontaneous electromyography (S-EMG, T-EMG), bulbocavernosus reflex (BCR) and train of four (TOF) monitoring. Loss of BCR responses during conus exposure and identification were resolved with multiple small pauses in manipulation throughout the procedure. T-EMG mapping aided in identification and avoiding the removal of nervous tissue.

Postoperatively the patient experienced some mild weakness in his left foot and leg that correlated with a significant amplitude drop in the left abductor hallucis TCeMEP. By the following day, the patient was almost back to preoperative baseline. The patient’s bowel and bladder function were preserved, consistent with final BCR recordings. The patient was discharged to rehabilitation postoperatively. Pathology results indicated glioblastoma.

This case study demonstrates the utility of a multimodality approach with bulbocavernosus reflex and urethral sphincter monitoring to optimize intraoperative data to the surgeon during conus tumor surgeries.

## Introduction

The conus medullaris is the tapered ending of the spinal cord that gives rise to the individual nerve roots flowing in the cauda equina through the vertebral foramen. Tumors in this region can instigate sensory and motor symptoms in the lower extremities as well as bowel and bladder dysfunction [1].

Intraoperative neurophysiological monitoring (IONM) is utilized to monitor neurological function of the lower limbs and distinguish nervous tissue from tumorous tissue during the resection. Multiple authors recommend the use of somatosensory evoked potentials (SSEPs), transcranial electrical motor evoked potentials (TCeMEPs), and spontaneous and triggered electromyography (S-EMG and T-EMG) as a multimodality approach to avoid new deficits [2-4]. Bulbocavernosus reflex (BCR), anal sphincter (AS), and urethral sphincter (US) recordings have also been suggested as valuable adjuncts to conus tumor monitoring [5-9].

## Case presentation

Patient history

The patient was a 75-year-old male who presented with progressive lower extremity weakness, pain in his legs bilaterally and loss of bowel and bladder control. Routine imaging revealed an intradural spinal cord tumor in the vicinity of T10-L2. Differential diagnoses included primary tumors of the spinal cord (ependymoma and astrocytoma), metastasis and lymphoma. The patient’s height was 1.8 m and weight was 86.8 kg. Informed patient consent was obtained prior to anesthesia administration.

Anesthesia

A total intravenous anesthesia regimen was utilized for this procedure with an infusion of propofol at 100 micrograms per kilogram per minute and sufentanil at 0.2 micrograms per kilogram per hour. The patient was kept warm with forced air warming blankets, and the mean arterial pressure was kept above 80 mmHg throughout the procedure. A small dose (20 milligrams) of rocuronium was administered for intubation and was completely worn off after positioning of the patient in a prone position with the arms on boards.

IONM

A certified neurophysiological intraoperative monitor performed the neurophysiological testing throughout the procedure [10]. IONM included the use of SSEPs, TCeMEPs, EMG, BCR, train of four (TOF), and electroencephalography (EEG) for depth of anesthesia.

SSEP adhesive surface pads were placed bilaterally over the ulnar nerves for stimulation (intensity: 20 mA, pulse width: 300 µs, repetition rate: 2.79 Hz). Following intubation, needle electrodes were placed at the posterior tibial nerve at the ankle and the pudendal nerve at the dorsal shaft of the penis. SSEPs could not be reliably obtained from the lower limbs or the pudendal nerve despite troubleshooting. Popliteal fossa recordings confirmed valid stimulation, but subcortical and cortical signals could not be acquired. Lower SSEP troubleshooting efforts included increasing the stimulation intensity to a maximum of 100 mA, increasing the pulse width, and the use of a variety of repetition rates. Popliteal fossa stimulation was then utilized, but did not resolve the absent lower SSEPs. SSEPs were recorded with subdermal needle electrodes at Erb’s point, the popliteal fossa, CP3, CP4, CPz, Cv5, and FPz.

**Figure 1:**
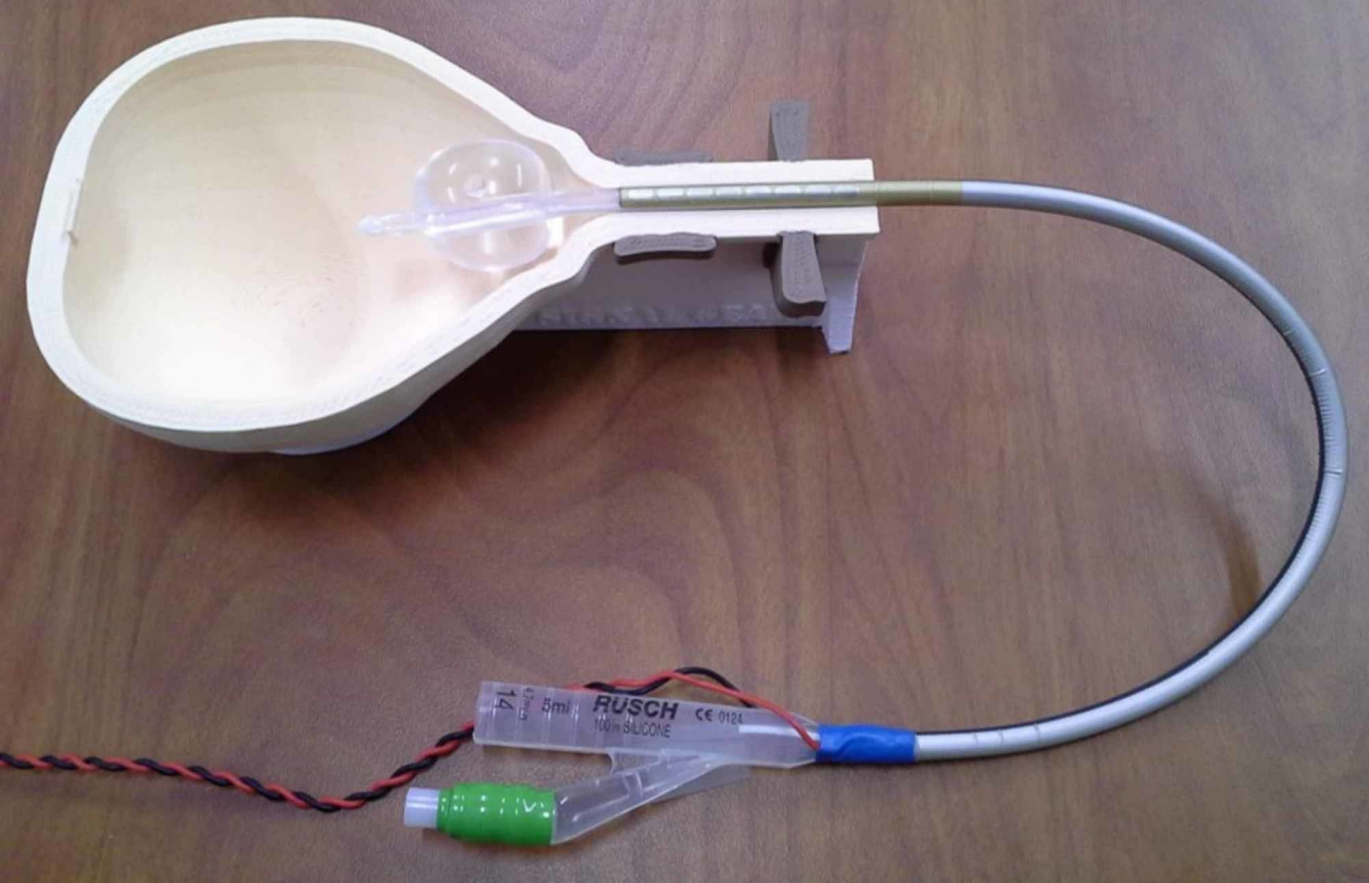
Image of bladder catheter with gold electrodes illustrating electrodes recording from the external urethral sphincter for electromyography and transcranial motor evoked potential recording collection (with permission from Signal Gear, Columbia, SC).

TCeMEPs were elicited with a linked quadripolar design from C1/M3 and C2/M4. Recording electrodes for TCeMEPs and EMG consisted of subdermal needle electrodes in the bilateral thenar muscles (TCeMEPs only), abdominis rectus, anal sphincter, adductor brevis, vastus medialis, gastrocnemius, and abductor hallucis. A urethral catheter with gold recording electrodes was placed in the external urethral sphincter (Figure 1).

A reliable four out of four twitches was obtained from the bilateral abductor hallucis muscles with stimulation of the posterior tibial nerves.

BCR stimulation of the pudendal nerve was achieved with central cathode needle electrodes placed at the dorsal base of the shaft of the penis below the pubic bone, and anode electrodes placed lateral of the cathodes (intensity: 20-30 mA, pulse count: 5, pulse width: 500 µs, interstimulus interval: 3.1 ms). BCR recording was achieved with 19 mm non-insulated straight needle electrodes inserted through the glabrous non-keratinized skin of the anal verge with two electrodes in each hemi-sphincter approximately 1 cm apart. All IONM was performed with a 32 channel Cascade Pro with Surgical Studio software by Cadwell Industries (Kennewick, WA).

Course of surgery

**Figure 2:**
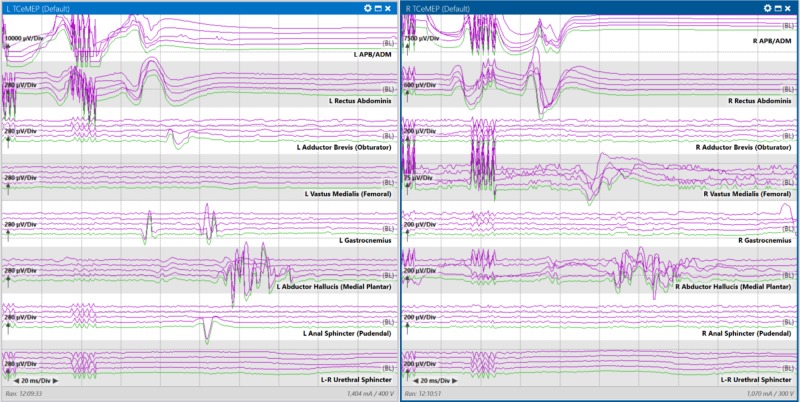
Baseline transcranial electrical motor evoked potential (TCeMEP) left and right side muscle recordings depicting multiple traces to illustrate reproducibility and variability among muscles (reliably obtained in the bilateral thenar, rectus abdominis and feet muscles). Left pane: Left side muscle recordings with transcranial stimulation; Right pane: Right side muscle recordings with transcranial stimulation.

At baseline, transcranial electrical motor evoked potentials (TCeMEPs) reproduced from the bilateral thenar muscles, rectus abdominis, and abductor hallucis muscles (Figure 2).

**Figure 3:**
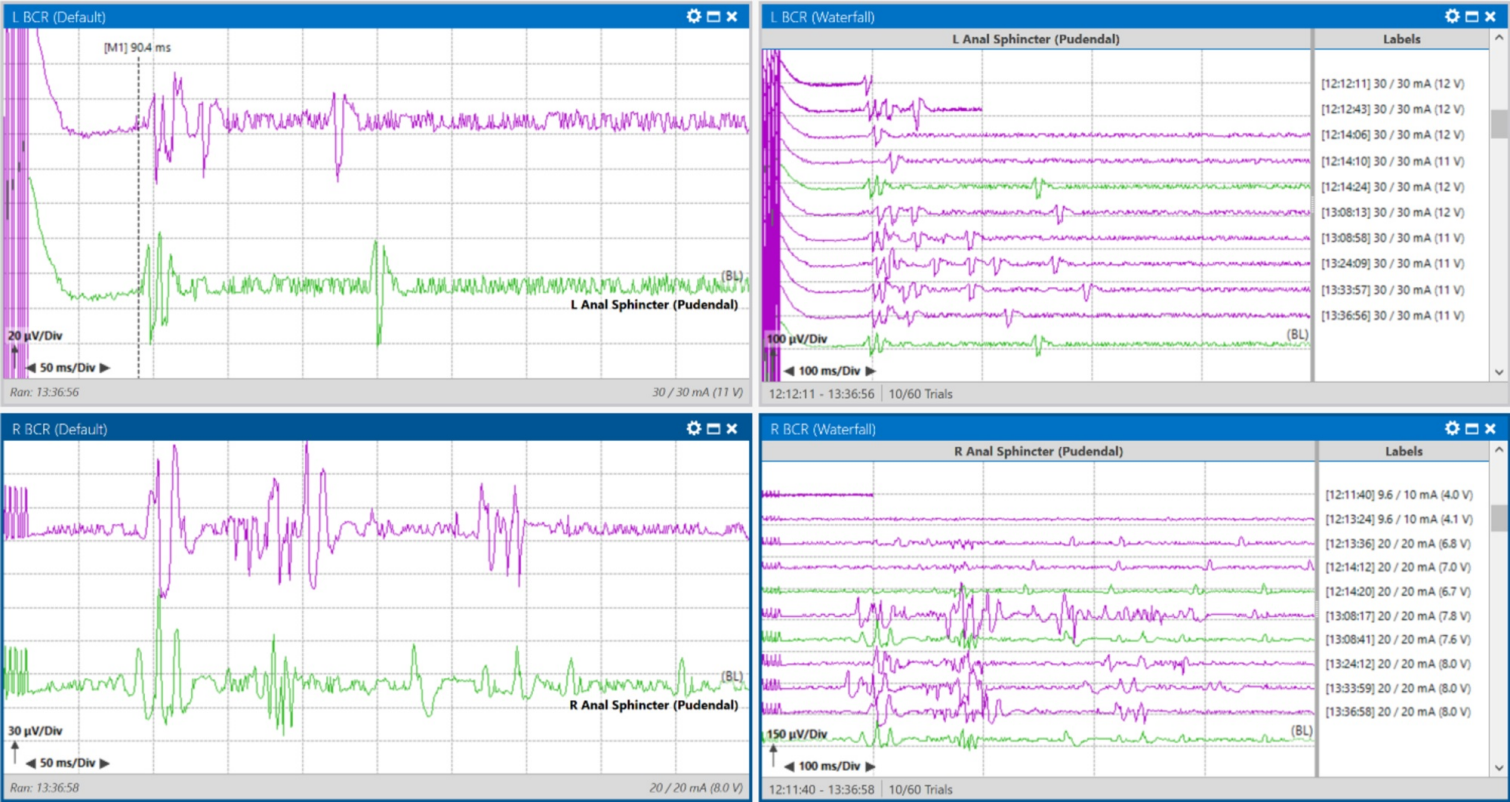
Baseline bulbocavernosus reflex (BCR) recordings taken from a patient with an intramedullary spinal cord tumor at T10-L2 were reliably obtained from the anal sphincter bilaterally. Right side images show a waterfall view of all traces collected before incision. Green traces indicate the trace chosen as a baseline, purple traces indicate all other traces that were acquired. Left upper pane: Average BCR response from left anal sphincter muscles. Right upper pane: Stack BCR response from left anal sphincter muscles. Left lower pane: Average BCR response from right anal sphincter muscles. Right lower pane: Stack BCR response from the right anal sphincter muscles.

BCR early responses were recorded at approximately 90 milliseconds reliably prior to a dural incision (Figure 3). BCR early responses are usually noted to fall within 30-35 ms, so an expanded time base was utilized [11].

SSEPs were not monitorable from the lower extremities at baseline despite significant troubleshooting, however, ulnar SSEPs were reliable with no remarkable changes throughout the procedure.

**Figure 4:**
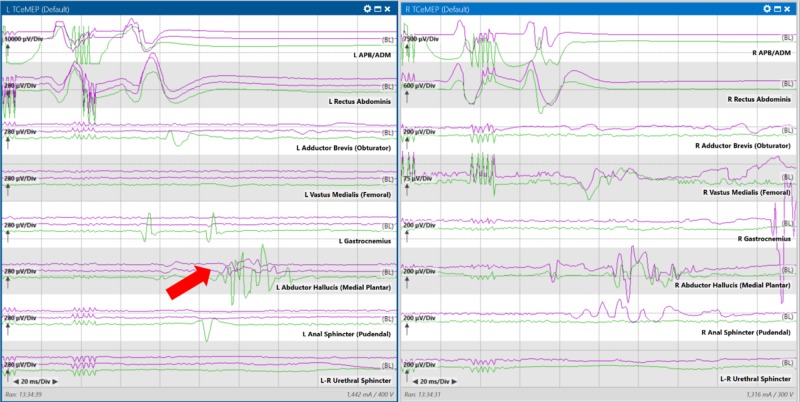
Reduction in the amplitude of the left abductor hallucis muscle transcranial electrical motor evoked potential recording (red arrow pointing to purple traces) from baseline (green traces) with the opening of the dura in a patient with intradural intramedullary spinal cord tumor at T10-L2. L APB/ADM = left abductor pollicis brevis and abductor digiti minimi; R APB/ADM = right abductor pollicis brevis and abductor digiti minimi Left pane: Compound muscle action potentials evoked from left side body muscles with transcranial stimulation; Right pane: Compound muscle action potentials evoked from right side body muscles with transcranial stimulation.

After opening the dura, a significant drop in the left abductor hallucis TCeMEP was identified and reported to the surgeon. The BCR responses remained stable (Figure 4).

**Figure 5:**
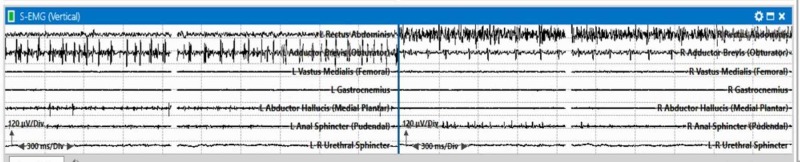
Spontaneous electromyography train activity was observed in multiple muscles during conus medullaris exposure in a patient with T10-L2 intradural intramedullary spinal cord tumor.

After 20 minutes of conus and cauda equina exposure and manipulation with preliminary T-EMG stimulation of the tissue, significant bilateral spontaneous EMG activity was observed (Figure 5).

**Figure 6:**
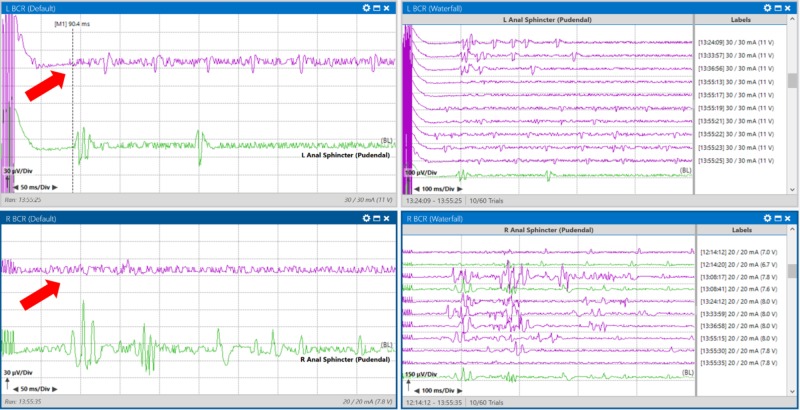
Loss of bilateral bulbocavernosus responses. Loss (purple traces), baseline (green traces) during manipulation at the conus. Left upper pane: Average BCR response from left anal sphincter muscles. Right upper pane: Stack BCR response from left anal sphincter muscles. Left lower pane: Average BCR response from right anal sphincter muscles. Right lower pane: Stack BCR response from right anal sphincter muscles.

After further manipulation, the bilateral BCR responses were lost (Figure 6).

**Figure 7:**
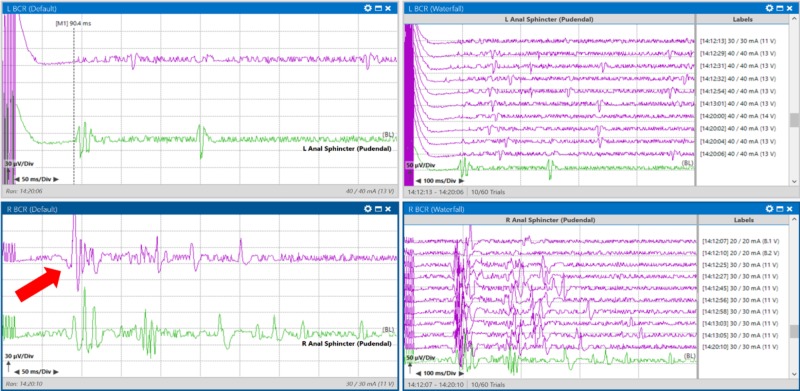
Recovery of the right bulbocavernosus reflex signal (red arrow pointing to purple trace) from baseline (green trace) with a nine-minute surgical pause. Left upper pane: Average BCR response from left anal sphincter muscles. Right upper pane: Stack BCR response from left anal sphincter muscles. Left lower pane: Average BCR response from right anal sphincter muscles. Right lower pane: Stack BCR response from right anal sphincter muscles.

The surgeon decided to remove all instruments from the surgical field and pause for BCR responses to recover. After nine minutes, the right BCR amplitude returned to baseline values (Figure 7).

**Figure 8:**
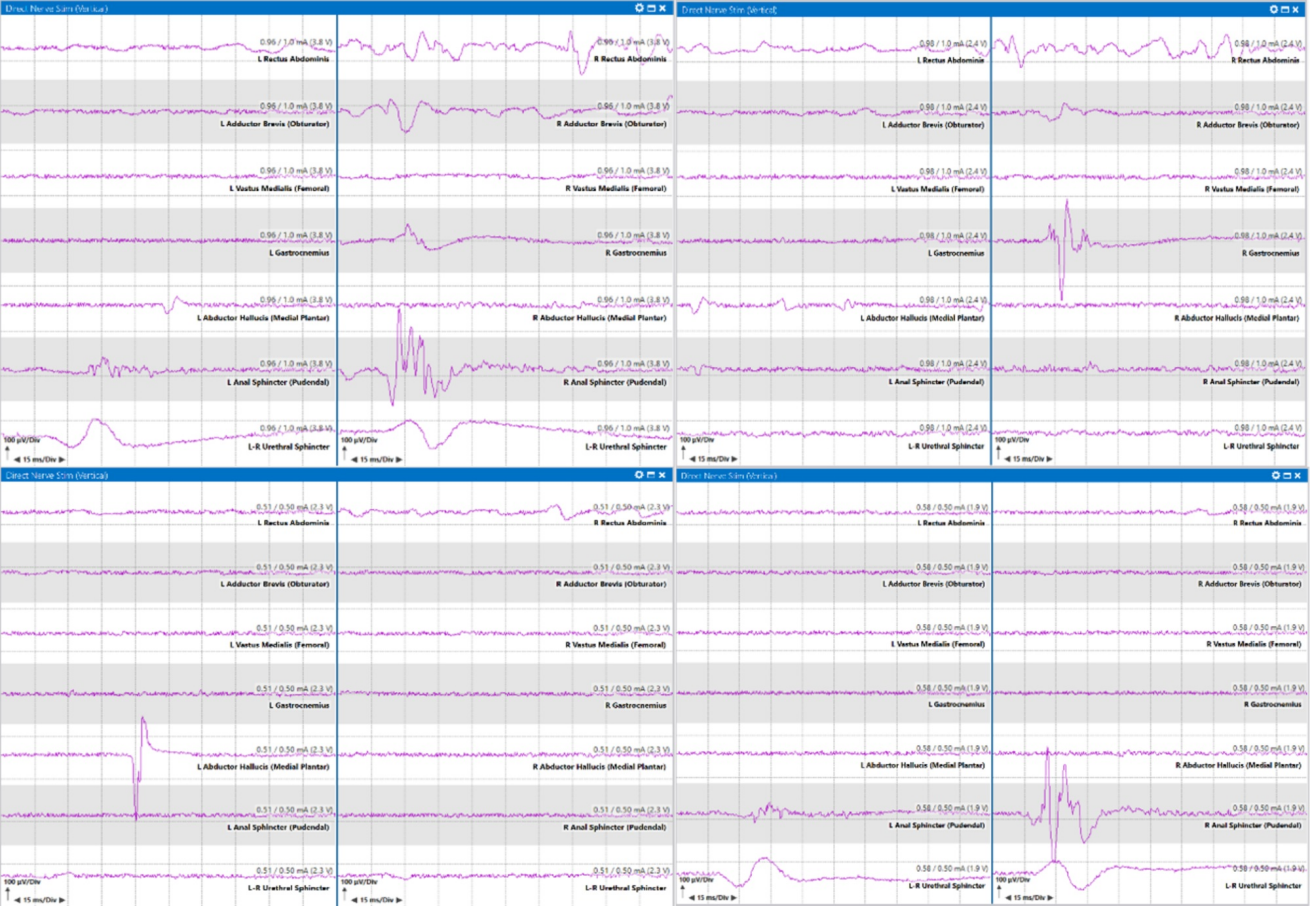
Examples of triggered electromyography mapping results of the conus medullaris and tumor with heavy anal sphincter and urethral sphincter representation in patient with intradural intramedullary spinal cord tumor at T10-L2. Left upper two panes: Muscle responses from conus stimulation identified in the left abductor hallucis, left anal sphincter, right rectus abdominis, right adductor brevis, right gastrocnemius, right anal sphincter and urethral sphincters at 1.0 milliampere; Right upper two panes: Muscle responses from conus stimulation identified in the right gastrocnemius at 1.0 milliampere; Left lower two panes: Muscle responses from conus stimulation identified in the left abductor hallucis at 0.5 milliamperes; Right lower two panes: Muscle responses from conus stimulation identified in the anal and urethral sphincters at 0.5 milliamperes.

The surgeon cautiously continued with a limited biopsy and T-EMG mapping to avoid removal of nervous tissue and resected small fragments to send to pathology. T-EMG responses were observed in the anal sphincter, urethral sphincter, abductor hallucis, and gastrocnemius muscles during mapping (Figure 8).

**Figure 9:**
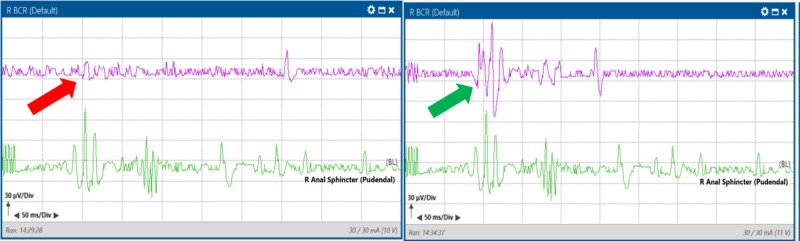
Loss (red arrow pointing at purple trace) with manipulation, and recovery (green arrow pointing at purple trace) from baseline (green traces) of the right bulbocavernosus reflex with a three-minute surgical pause during biopsy of a patient with T10-L2 intradural intramedullary spinal cord tumor. Left pane: Loss of right bulbocavernosus reflex response at 14:29 (green is baseline) Right pane: Recovery of right bulbocavernosus reflex response at 14:34 after a surgical pause

With further manipulation and biopsy, the right BCR was lost again, and the surgeon chose to avoid this portion of tissue. The response returned three minutes later (Figure 9).

After multiple very small specimens were obtained, the surgeon and pathologist agreed there was diagnostic tissue. Given the lack of tumor differentiation from normal tissue, the surgeon elected to end the surgery and wait for final pathological diagnosis.

**Figure 10:**
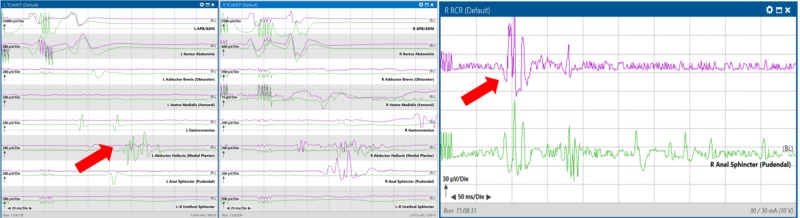
Final transcranial electrical motor evoked potential (TCeMEP) and bulbocavernosus reflex (BCR) responses recorded at the end of surgery demonstrated a small response in the left foot promising for recovery (left red arrow pointing to the purple trace, the green trace is the baseline response), and a response consistent with the baseline for the right BCR (right red arrow pointing to the purple trace, the green trace is the baseline response). Left pane: Left TCeMEP responses; Middle pane: Right TCeMEP response; Right pane: Right BCR responses.

At closing of the skin, the left abductor hallucis TCeMEP was present but remained severely reduced in amplitude and complexity. The right BCR was consistent with the baseline (Figure 10).

Postoperative outcome

Postoperatively the patient experienced some mild weakness in his left foot and leg, consistent with the amplitude change in the left abductor hallucis TCeMEP. By the following day, the patient was almost back to preoperative baseline functioning. The patient’s bowel and bladder function was preserved and the patient was discharged to rehabilitation two days postoperatively. Tissue was sent to the Mayo clinic for review and was determined to be a high-grade infiltrating glial neoplasm consistent with glioblastoma multiforme.

## Discussion

The bulbocavernosus reflex monitors the integrity of the conus reflex circuitry indicating intact sphincter control. The BCR is an oligosynaptic reflex that is mediated through the S2-S4 spinal cord segments. It is achieved by genital stimulation and recording from the pelvic floor or anal sphincter muscles. It has frequently been used as a measure of spinal cord shock after spinal cord injury. The BCR monitors pudendal nerve somatic sensory and motor fibers, the cauda equina, Onuf’s nucleus in the conus medullaris, and the parasympathetic nervous system by proxy [7, 11-14]. Urethral sphincter monitoring is achieved by the placement of a catheter with gold electrodes that rest at the level of the external urethral sphincter [8-9, 15].

Case studies by Skinner et al. have demonstrated the utility of the BCR in guiding dissection, suturing and use of electrocautery, and more case studies need to be provided to correlate neurophysiological data with clinical outcomes [11].

Conus tumor biopsy and resection is a surgery with a high risk of damage to the motor and sensory pathways to the lower limbs as well as to bowel and bladder function. Manipulation, laser, and electrocautery usage can significantly impact neuronal functioning. Use of BCR and urethral T-EMG during the resection aids in identification of neural tissue and the minimization of neural injury from over manipulation. Taking surgical pauses when significant changes or degradations in neurophysiological data occur may prevent permanent injury and allow the surgeon to complete a fuller dissection with less neurological distress.

## Conclusions

This case study demonstrates the utility of a multimodality approach with bulbocavernosus reflex and urethral sphincter monitoring to optimize intraoperative data to the surgeon during conus tumor surgeries that can act as a barometer for nervous system functional tolerance. An intradural intramedullary spinal cord tumor resection carries a very high risk of damaging the spinal cord and nerve roots due to the proximity of vital nervous tissues. In this patient, real-time multimodality IONM was useful for the early identification of spinal cord injury during the surgical procedure. With the use of continuous neuromonitoring including the bulbocavernosus reflex modality, the surgery was paused promptly with the identification of changes, thus minimizing the duration of spinal ischemia. We highly recommend utilizing continuous multimodality IONM, including SSEP, TCeMEP, BCR, and EMG during cauda equina and conus tumor surgery by a highly trained technologist and/or neurophysiologist.
